# Ultrasound-Guided Selective Glossopharyngeal Nerve Block for Severe Gag Reflex During Dental Treatment: A Report of Two Cases

**DOI:** 10.7759/cureus.54725

**Published:** 2024-02-22

**Authors:** Yuki Kojima, Daisuke Oiwa

**Affiliations:** 1 Department of Dental Anesthesiology, Asahi General Hospital, Asahi, JPN; 2 Department of Dental Anesthesiology and Perioperative Management, Hinode Makomanai Dental Hospital, Sapporo, JPN

**Keywords:** gagging, dentistry, sedation, glossopharyngeal nerve, dental care, nerve block

## Abstract

The ultrasound-guided selective glossopharyngeal nerve block (UGSGNB) has been developed as an approach to overcome the drawbacks of the conventional glossopharyngeal nerve block. The UGSGNB may be performed when a gag reflex occurs during dental treatment. Case 1 involved a 67-year-old man with a medical history of cervical spondylosis and dilated cardiomyopathy. Dental treatment with conscious sedation and the UGSGNB was performed three times. Case 2 involved a 25-year-old woman who was scheduled for dental treatment under general anesthesia because of dental phobia and gagging. Because the patient experienced severe tooth pain and desired urgent treatment, anesthesia was induced with intravenous sedation and the UGSGNB. In both cases, treatments were completed without intraoperative gagging or any complications. Our observations indicate that the UGSGNB can suppress the gag reflex during dental treatment; it may allow surgeons to avoid inducing general anesthesia and deep sedation in patients with a severe gag reflex.

## Introduction

A gag reflex is an involuntary muscle contraction at the back of the throat. It prevents objects in the oral cavity from entering the throat in instances other than during normal swallowing and helps prevent choking [1]. People with a hypersensitive gag reflex encounter difficulties in several situations, such as while swallowing a pill and during dental procedures and endoscopy. In cases wherein the gag reflex is particularly severe, medical treatment may be difficult without general anesthesia or deep sedation [2-4].

Garg et al. reported that a glossopharyngeal nerve block (GNB) may be performed during dental procedures in patients with an exaggerated gag reflex [5]. Conventional GNB by the landmark technique is performed with either an intra-oral or an extra-oral approach. However, conventional GNB is associated with a risk of upper airway obstruction. One study reported that a lot of local anesthetics were sufficient for blocking the vagus nerves proximal to where the recurrent laryngeal nerves and the hypoglossal nerves branch off [6]. The ultrasound-guided selective glossopharyngeal nerve block (UGSGNB) has been developed recently as an approach to overcome the drawbacks of the conventional GNB [7]. Herein, we report the cases of two patients with a severe gag reflex who underwent dental treatment under sedation and the UGSGNB.

## Case presentation

Case 1

The patient was a 67-year-old man (height: 185 cm, weight: 93 kg) with a medical history of cervical spondylosis and dilated cardiomyopathy. He had a severe gag reflex, which led to difficulties while undergoing dental treatments and gastroscopy. He was referred for deep sedation because of the gag reflex; however, the blood pressure decreased significantly following propofol (20 mg) administration for sedation. Therefore, the dental treatment was canceled. We planned to perform the UGSGNB to suppress the gag reflex under sedation with midazolam. 

**Figure 1:**
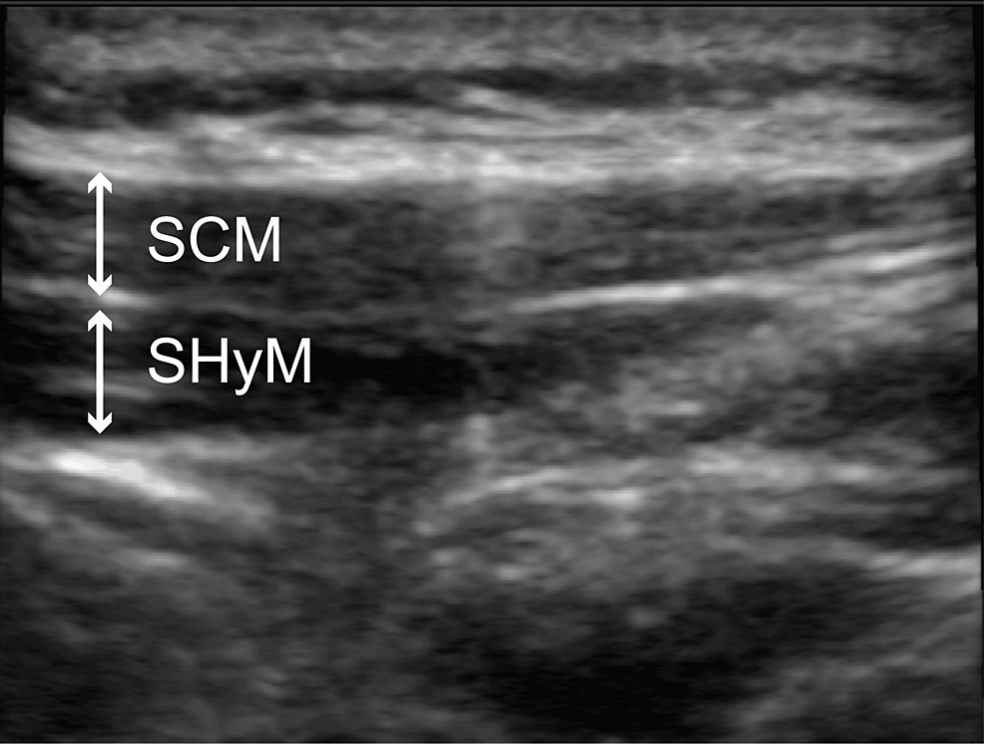
Ultrasonographic images obtained before ultrasound-guided selective glossopharyngeal nerve block in Case 1 After the sternocleidomastoid muscle is identified, a linear ultrasound probe is set parallel to the sternocleidomastoid muscle running just caudal to the mandibular ramus. SCM, sternocleidomastoid muscle; SHyM, stylohyoid muscle

**Figure 2:**
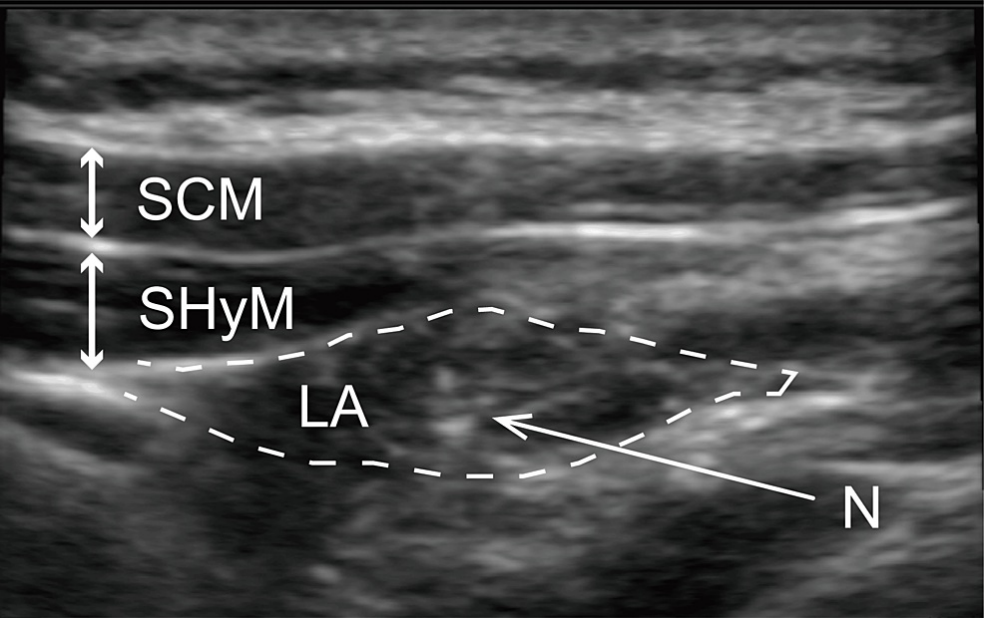
Ultrasonographic images obtained during ultrasound-guided selective glossopharyngeal nerve block in Case 1 A 25-gauge, 25 mm needle is inserted deeply under the stylohyoid muscle through the sternocleidomastoid muscle via an out-of-plane approach. The spread of the local anesthetic is confirmed. SCM, sternocleidomastoid muscle; SHyM, stylohyoid muscle; LA, local anesthetic; N, needle tip

After securing the intravenous line with a 22-gauge cannula, we administered midazolam (2 mg) under standard monitoring with the following devices: electrocardiography electrodes, pulse oximeter, and blood pressure monitor. The UGSGNB was performed as follows: the patient turned their face toward the clinician, and an ultrasound probe was inserted into their neck to monitor the sternocleidomastoid and stylohyoid muscles (Figure 1). A 23-gauge, 25 mm needle was inserted deeply under the stylohyoid muscle through the sternocleidomastoid muscle using an out-of-plane approach. An ultrasound-guided nerve block was performed bilaterally, and 1.5 mL of 1% lidocaine was administered to each side (Figure 2). The swallowing reflex occurred normally, while the patient did not have any gag reflexes during treatment. In total, the patient underwent dental treatment with conscious sedation and the UGSGNB three times; in all instances, the treatment was completed without any complications. The first operation time was one hour and four minutes, and the first anesthesia time was one hour and 34 minutes. The patient stated that he had no unpleasant memories, and his satisfaction level was very high. 

Case 2

**Figure 3:**
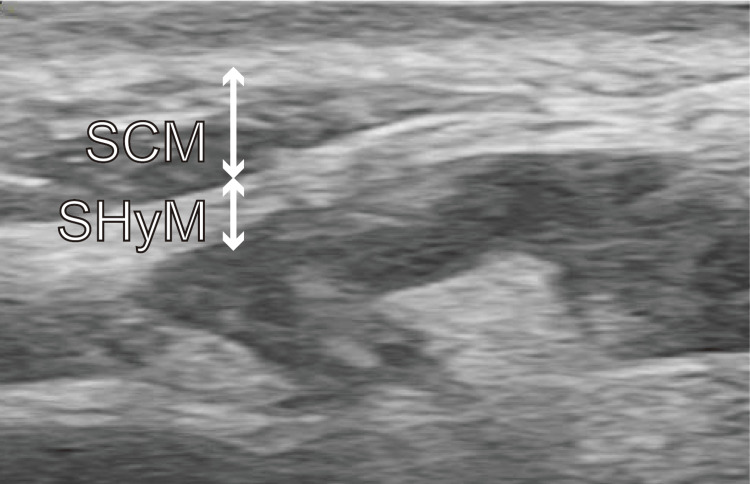
Ultrasonographic images obtained before ultrasound-guided selective glossopharyngeal nerve block in Case 2 The sternocleidomastoid and stylohyoid muscles are observed behind the mandibular ramus. SCM, sternocleidomastoid muscle; SHyM, stylohyoid muscle

The patient was a 25-year-old woman (height: 156 cm, weight: 46 kg) who was referred for general anesthesia because of dental phobia, gag reflex, and trismus. The patient was scheduled for a pulpectomy and tooth extraction for pulpitis and mandibular wisdom tooth periodontitis, respectively. Because the patient experienced severe pain and desired urgent treatment, we decided to induce anesthesia with intravenous sedation and an ultrasound-guided inferior alveolar nerve block (UGIANB) and UGSGNB. The patient was induced after being attached to the following monitoring devices: electrocardiography electrodes, pulse oximeter, and blood pressure monitor. After establishing an intravenous line with a 22-gauge cannula, propofol treatment was started using target control infusion at a target blood concentration of 1.2 µg/mL. After confirming that the patient’s anxiety had decreased, the UGIANB was performed on the left side, and 5 mL of 0.375% levobupivacaine was administered to this side (Figure 3). Thereafter, the UGSGNB was performed on the left side, and 1.5 mL of 1% lidocaine was administered to this side. After five minutes, we confirmed that touching the pharynx did not induce a gag reflex; thus, we started the procedure. Dental treatment was performed with conscious sedation and without any intraoperative gag reflex or complications. The operation time was 40 minutes, and the anesthesia time was 54 minutes. The patient stated that she had no unpleasant memories and that she was highly satisfied with the procedure.

## Discussion

This report describes the cases of two patients with a severe gag reflex that were managed with the UGSGNB. In both cases, we were able to successfully control the gag reflex and perform dental treatments without complications. Various treatments are available for severe gag reflex [8,9]. This reflex can be suppressed using behavioral techniques and relaxing music. Applying lidocaine to the posterior pharyngeal wall is also effective during short-term treatments. Furthermore, it has become a common practice to control the gag reflex with intravenous sedation during dental treatments and examinations [1,10]. Deep sedation may lead to complications in cases of a sedative overdose; therefore, strict control is required. The risk of respiratory and circulatory depression persists if large doses of sedatives are administered to control a severe gag reflex [11,12]. The UGSGNB offers several advantages over traditional glossopharyngeal nerve blocks; however, since the UGSGNB is a compartment block, it is necessary to perform a cadaver study to confirm proper infiltration of local anesthetics. It should also be noted that the target area is very shallow; the amount of injected local anesthetic is small; and the effects of lidocaine, when used as a local anesthetic, wear off in approximately an hour [7]. Based on this case report alone, it cannot be established whether the same effect can be expected in all cases.

## Conclusions

The UGSGNB may be an effective option for patients who are unable to undergo dental treatment due to a severe gag reflex. It allows for safe anesthesia management because the amount of used sedatives can be reduced. However, the safety and efficacy of the UGSGNB require further verification through technical development and clinical research. It is particularly important to determine the appropriate local anesthetic dose through cadaver studies.
